# Preoperative Embolization in Tandem with Surgical Resection for Cerebral Arteriovenous Malformations

**DOI:** 10.7759/cureus.2042

**Published:** 2018-01-08

**Authors:** Richa Thakur, Ali S Haider, Ashley Thomas, Steven Vayalumkal, Umair Khan, Tijani Osumah, Kyle Doughty, Sam Finn, Kennith F Layton

**Affiliations:** 1 Texas A&M College of Medicine; 2 School of Medicine, St. George's University; 3 Department of Urology, Mayo Clinic; 4 Department of Neurosurgery, Baylor University Medical Center; 5 HSS Management; 6 Department of Radiology, Baylor University Medical Center

**Keywords:** arteriovenous malformation, embolization, preoperative

## Abstract

A number of treatment options are available for cerebral arteriovenous malformations (AVMs) including surgical resection, stereotactic radiosurgery, and endovascular embolization. Endovascular embolization may be used pre-operatively to reduce the size of large AVMs and thus reduce surgical complications. Here we present two patients who successfully underwent preoperative embolization of their AVMs and subsequent surgery. Preoperative embolization is a viable option for AVMs to reduce complications and improve patient outcomes.

## Introduction

Cerebral arteriovenous malformations (AVMs) are congenital vascular abnormalities that may cause intracranial hemorrhage, headaches or seizures. AVMs have direct connections from the arterial system to the venous system without an intermediate capillary bed. These high-flow lesions are responsible for 2-3% of symptomatic hemorrhages [1]. When left untreated, AVMs have an annual 2-4% rate of hemorrhaging [2]. Treatment options include surgical excision, stereotactic radiosurgery, endovascular embolization or multimodality treatment using a combination of these methods. While surgical excision has higher rates of cure, radiation therapy can still be used with cure as a goal of treatment in high surgical risk patients. Although radiation therapy can result in complete cure up to 80% of patients, complications include necrosis, increased neurologic defects, seizures, as well as a theoretical risk of hemorrhaging in the latency period [3]. In order to assist with the surgical approach in some patients, endovascular embolization can be used to reduce complications and increase the success rate of surgical excision by decreasing the size of large AVMs and occluding collateral vessels [4]. Presented below are two cases of cerebral arteriovenous malformations that were treated successfully with preoperative embolization.

## Case presentation

Case 1

**Figure 1:**
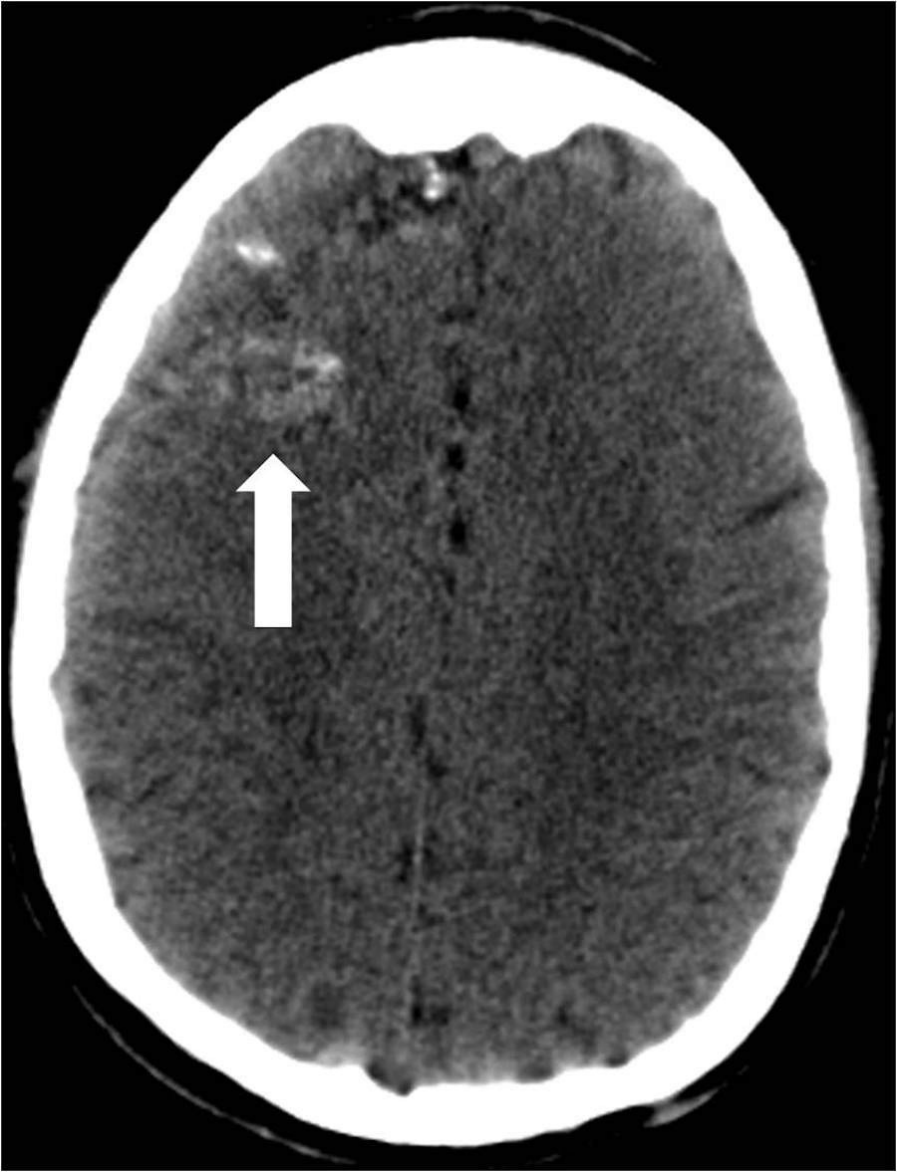
Computed tomography of the head reveals a right frontal lobe arteriovenous malformation with scattered calcifications (arrow).

A 26-year-old male with a history of seizures presented to an outside hospital (OSH) with a new onset seizure. The patient denied drinking alcohol or recreational drug abuse. He became more alert by the time he arrived at the OSH. Besides the initial seizure and a left frontal scalp hematoma secondary to seizure-related trauma, the patient was otherwise asymptomatic. He was initially treated with phenytoin. Computed tomography (CT) of the head at the OSH showed an AVM that involved the right frontal lobe roughly measuring 4.8 cm by 6.3 cm (Figure 1).

**Figure 2:**
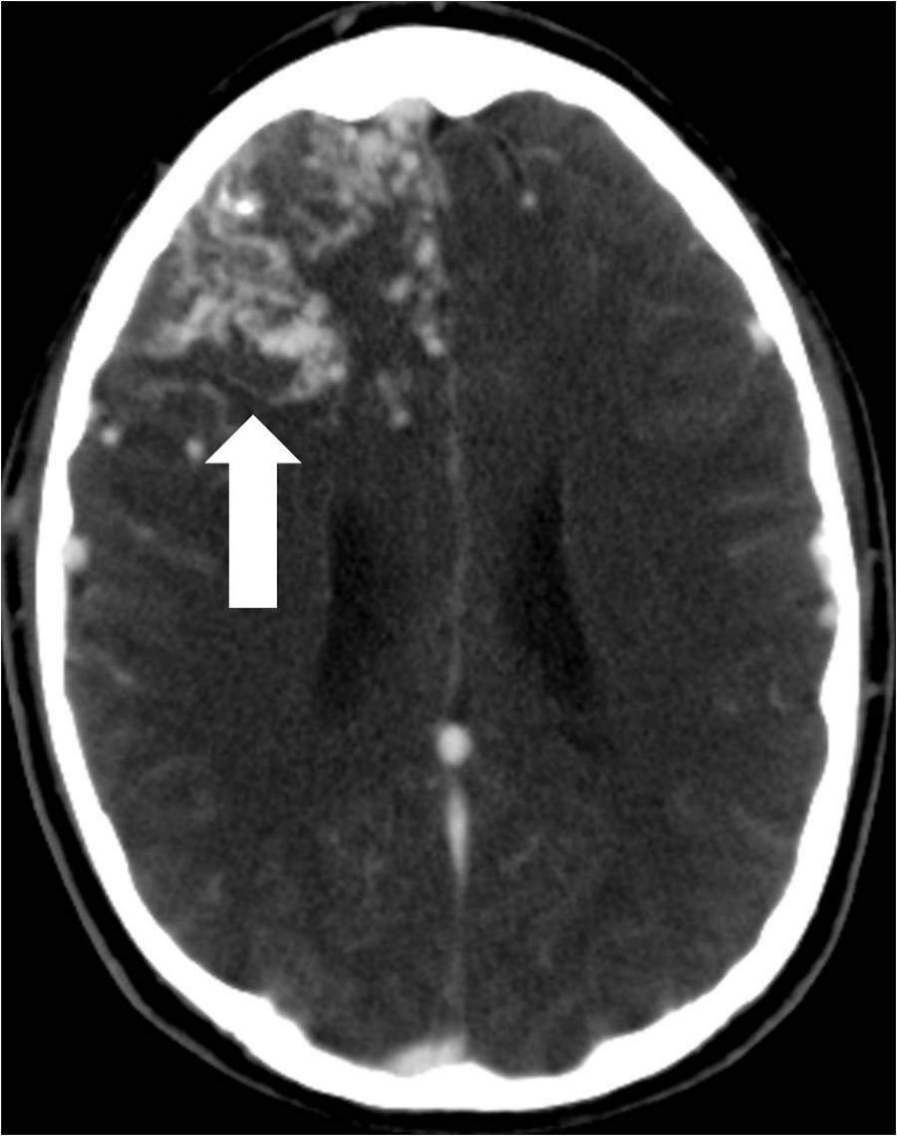
Computed tomography angiogram of the head shows the large enhancing right frontal lobe arteriovenous malformation nidus (arrow).

He was then transferred for a higher level of care to a high-volume Comprehensive Stroke Center. Cerebral angiography showed a non-ruptured left frontal AVM that was fed by anterior and middle cerebral artery branches (Figure 2).

**Figure 3:**
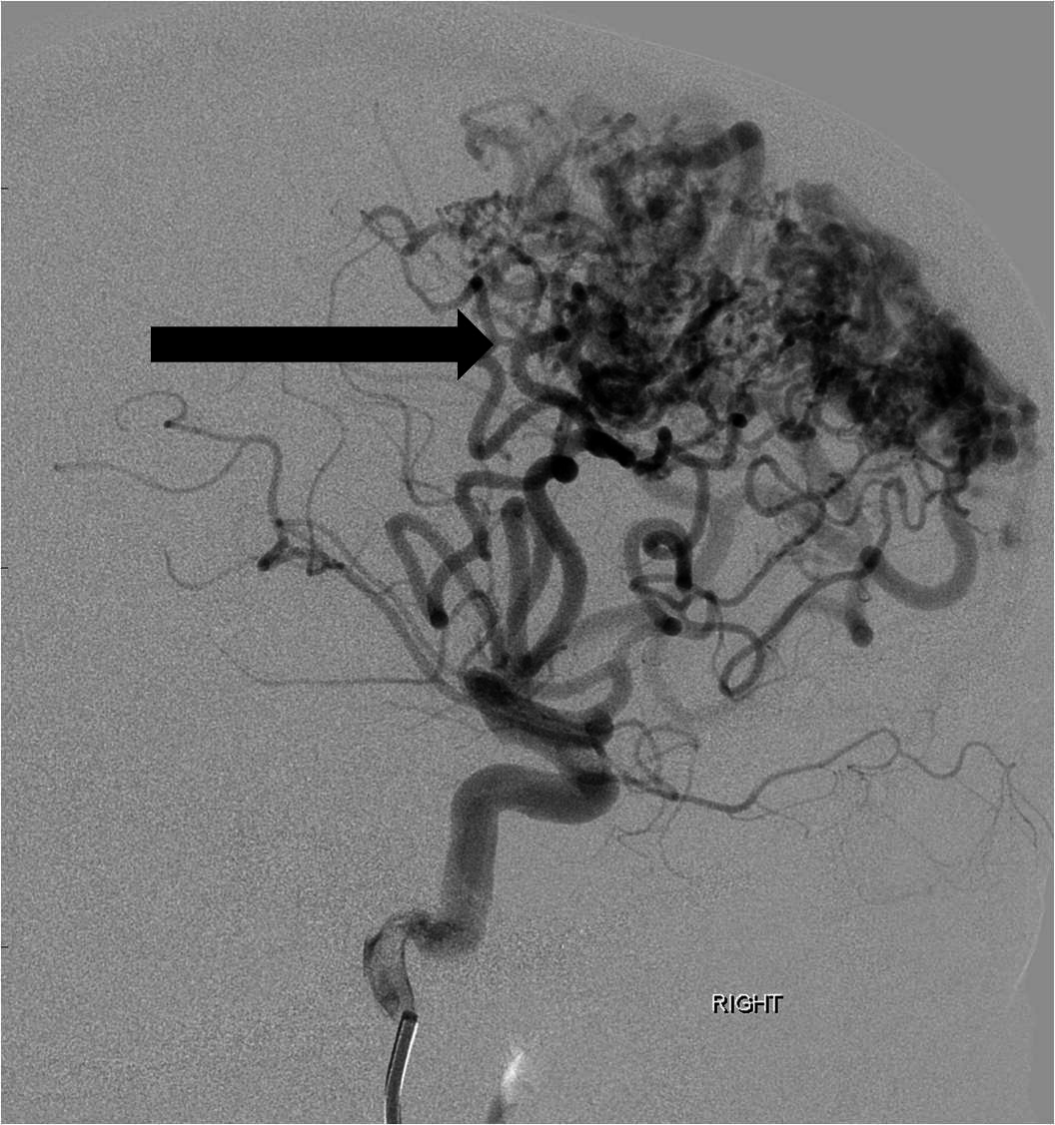
Lateral projection angiographic image from a right internal carotid artery injection shows a large arteriovenous malformation nidus (arrow) fed by multiple enlarged feeding anterior and middle cerebral artery branches. "Right" indicates the patient's right side.

He was scheduled for a pre-operative embolization the following week in anticipation of subsequent surgical resection. Under general anesthesia, four separate arterial feeding pedicles from the right anterior cerebral artery were catheterized (Figure 3).

**Figure 4:**
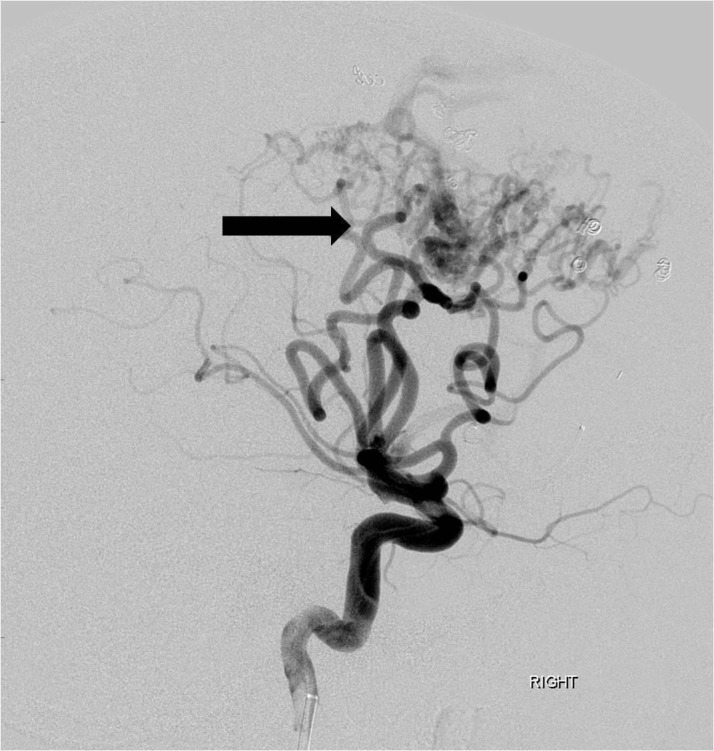
Following embolization of multiple anterior cerebral artery feeding branches with particles, gel foam and coils, there is marked reduction in size and flow dynamics of the arteriovenous malformation (arrow). "Right" indicates the patient's right side.

Each pedicle was treated with a combination of polyvinyl alcohol (PVA) particles (ranging in size from 250 to 500 microns), gelfoam torpedoes, and platinum coils of varying lengths and sizes. A post-embolization arteriogram demonstrated greater than 80% reduction in the overall angiographic volume of the arteriovenous malformation nidus (Figure 4).

**Figure 5:**
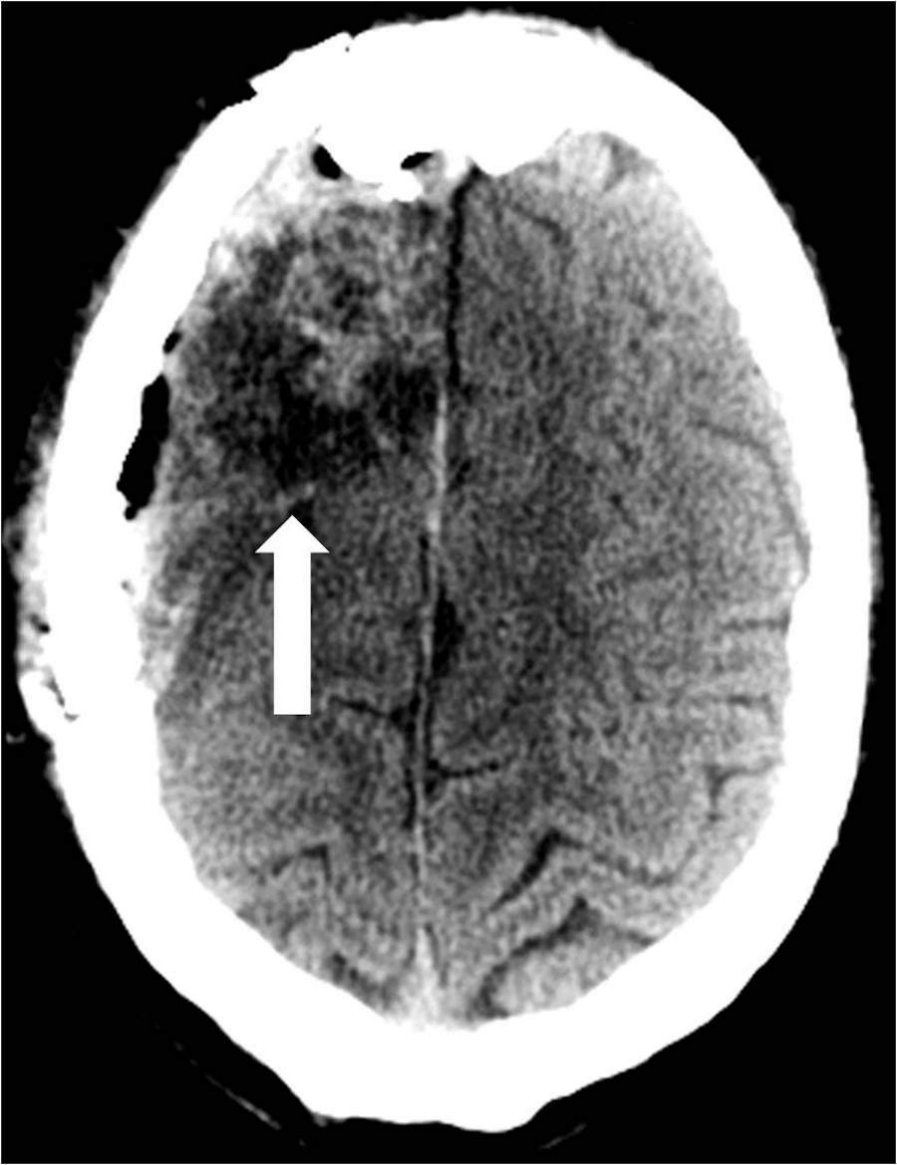
Head computed tomography on postoperative day three reveals expected changes of a right frontoparietal craniotomy with a resection cavity in the right frontal lobe (arrow).

The right middle cerebral artery feeding pedicles were not embolized due to their superficial location and ability to secure these pedicles upon initial surgical approach. The following day, the patient underwent a right frontoparietal craniotomy to resect the AVM. A subsequent postoperative head CT revealed no complications (Figure 5).

The patient's hospital course was relatively uncomplicated and he was discharged four days after the craniotomy.

Case 2

**Figure 6:**
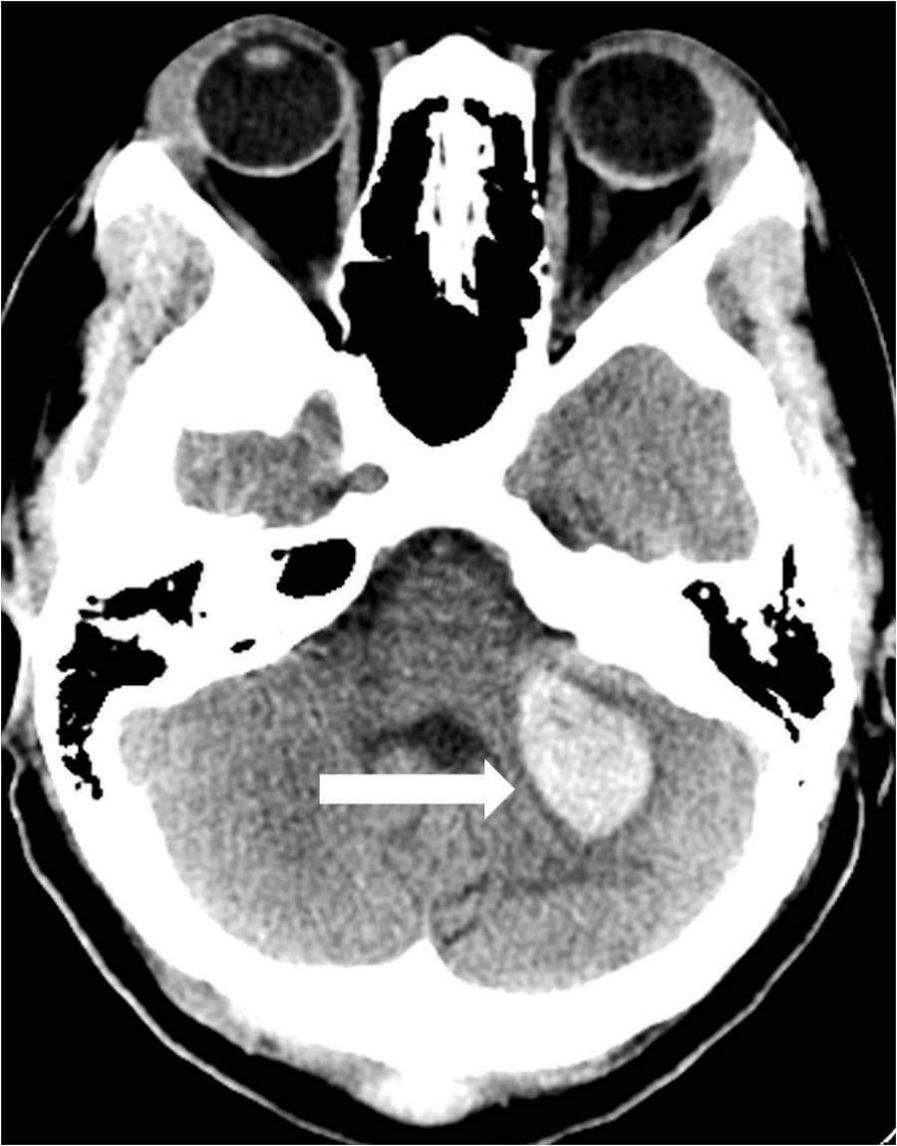
Initial head computed tomography reveals an acute intraparenchymal hemorrhage in the left cerebellar hemisphere (arrow).

A 39-year-old female with a history of posterior fossa AVM, AIDS, and hypertension presented with headaches, dizziness, blurry vision, and left-sided facial numbness. She had a previous history of hemorrhage from an AVM in the posterior fossa which was treated with a partial embolization two years prior. She was scheduled for a resection of the AVM at that time but was lost to follow up. Physical exam showed no abnormalities on admission. CT scan of the head showed an acute parenchymal hemorrhage in the inferior left cerebellum (Figure 6).

**Figure 7:**
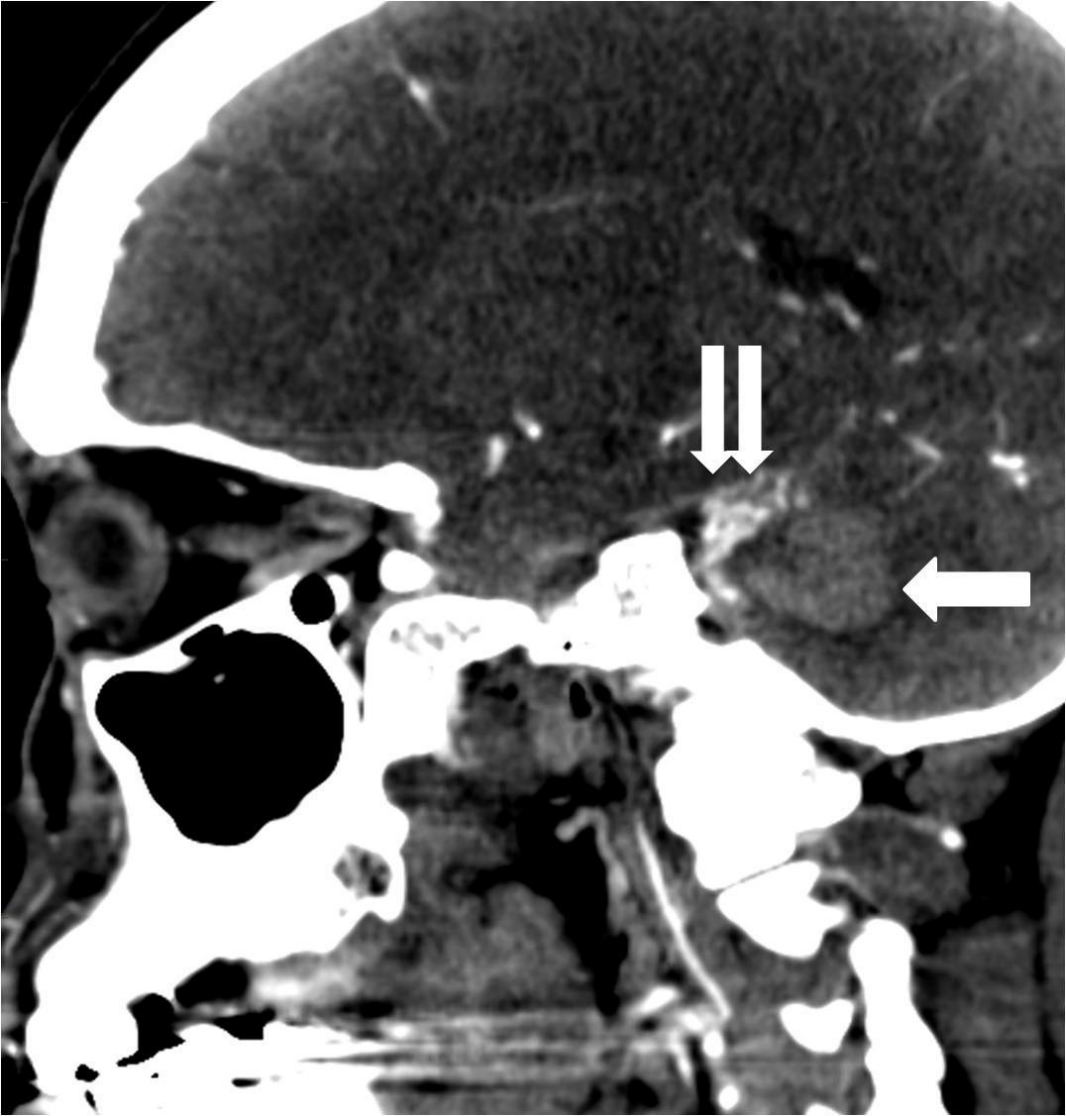
Sagittal reconstruction image from a computed tomography angiogram demonstrates the cerebellar parenchymal hematoma (arrow) and the adjacent arteriovenous malformation (double arrow).

CT angiogram (CTA) showed a left posterior fossa vascular malformation that had remained largely unchanged from the prior admission two years earlier (Figure 7).

**Figure 8:**
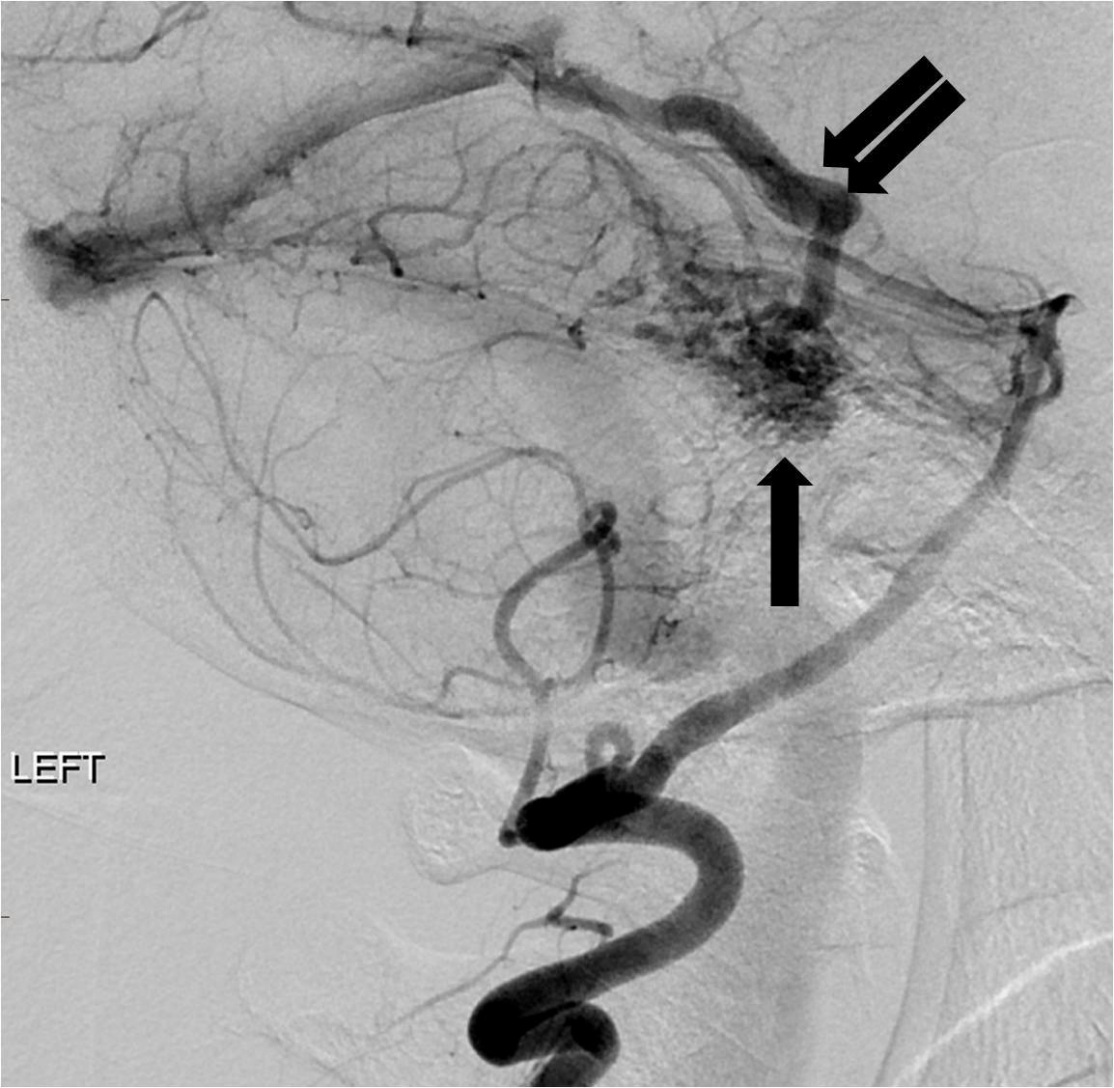
Lateral projection angiogram from a left vertebral artery injection reveals a compact arteriovenous malformation nidus in the posterior fossa (arrow) with an enlarged early draining vein (double arrow). "Left" indicates the patient's left side.

The patient was admitted for further workup of the intracranial hemorrhage. Cerebral angiography on day two of admission showed an AVM measuring 1 cm by 2 cm predominantly supplied by the left superior cerebellar artery as well as the left anterior inferior cerebellar artery (Figure 8).

**Figure 9:**
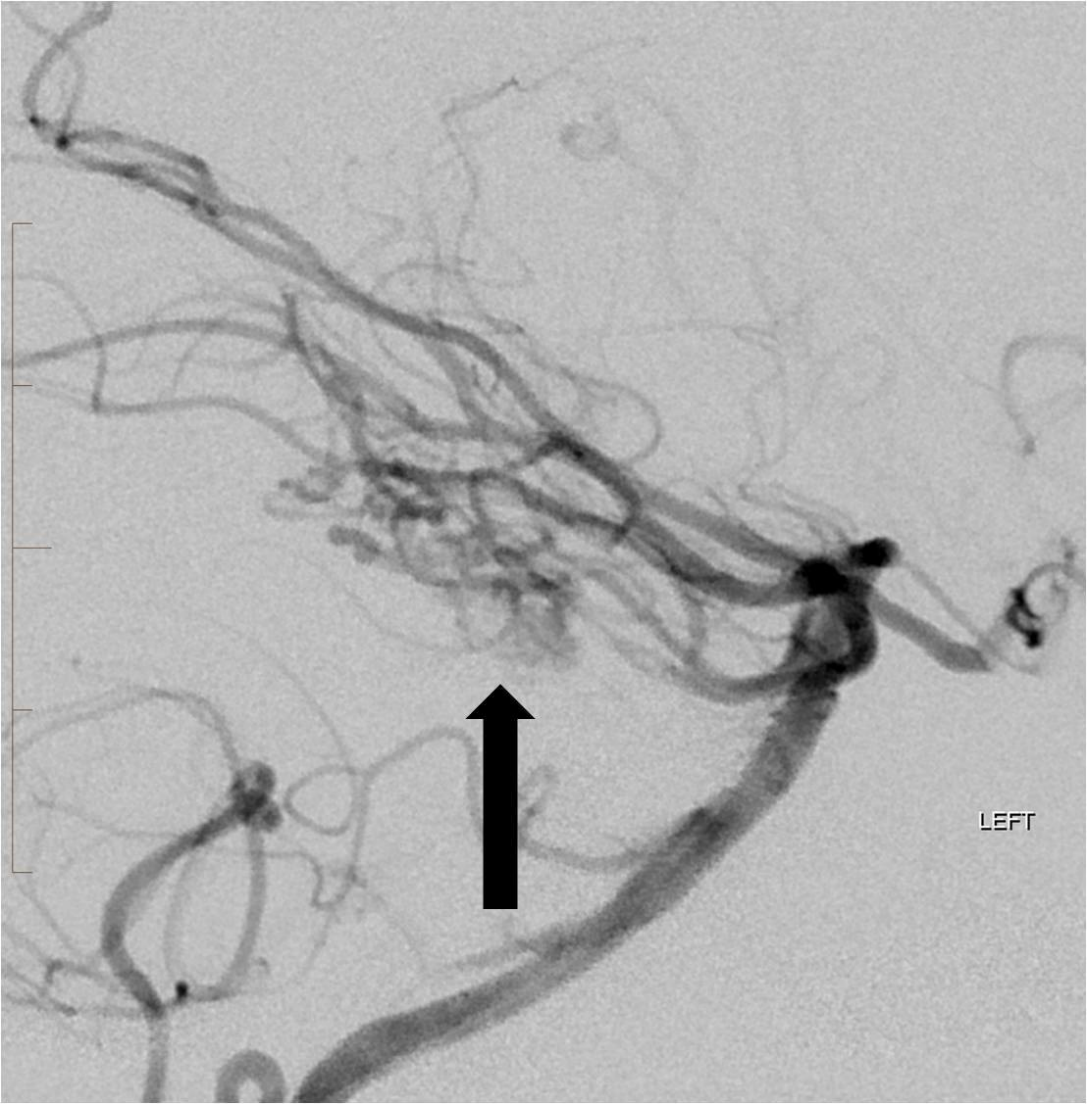
Following embolization of the superior cerebellar and anterior inferior cerebellar artery branches to the arteriovenous malformation, there has been marked reduction in the size and flow dynamics of the arteriovenous malformation nidus (arrow). "Left" indicates the patient's left side.

She was scheduled for pre-operative embolization and surgery the following week. Under general anesthesia, a 5-French catheter was advanced into the left vertebral artery where digital subtraction angiography (DSA) was performed. Over a microwire, a microcatheter was then advanced into the left superior and anterior inferior cerebellar arteries near the nidus of the AVM. A mixture of 250-355 micron PVA particles was then injected carefully under fluoroscopic observation until there was visible slowing of antegrade flow in each of the branches. Approximately 75% reduction in volume of the AVM was achieved and there was a marked reduction in the flow dynamics (Figure 9).

**Figure 10:**
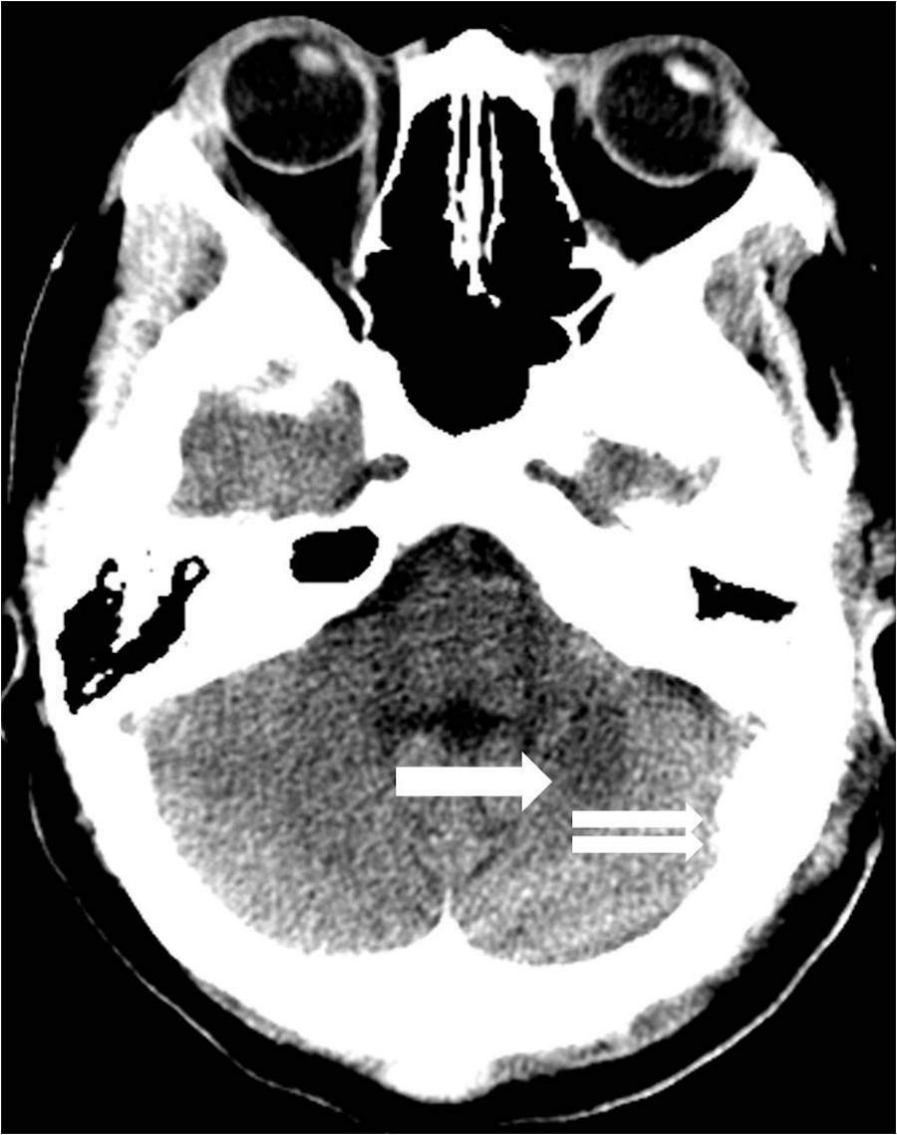
Postoperative head computed tomography two weeks after surgery reveals a focal area of encephalomalacia where the hematoma used to reside (arrow). Subtle changes from a left retromastoid craniotomy are evident (double arrow).

The following day, the patient underwent a left suboccipital craniotomy to resect the AVM. Postoperative head CT revealed no complicating features (Figure 10).

The patient's hospital course was relatively uncomplicated and she was discharged home 12 days after the craniotomy.

## Discussion

The primary treatment goal for cerebral AVMs is to obliterate the entire malformation to reduce the risk of hemorrhage. Secondary goals include seizure control, amelioration of headaches, and improving visual deficits. Many single-institution studies have failed to show a difference in outcomes based on single or multimodality approaches for cerebral AVMs [5]. Since clinical studies have not shown a consistent advantage to using one approach over the other, the neurosurgeon must choose an approach that maximizes benefits while minimizing adverse outcomes. In situations when endovascular cure is not possible, partial embolization can still be used for symptom management and to improve outcomes with surgical resection [6]. Pre-operatively, embolization can be used to decrease the volume of an AVM to make it more amenable for surgical resection. Although embolization seldom cures AVM in their entirety as opposed to radiation or surgical resection, it is still a viable alternative in several situations. When patients are not stable enough for neurosurgery during a hemorrhagic episode, embolization can be used as a palliative approach to prevent rebleeding until the patient is stable enough to proceed with other options [7]. In cases where the AVM is not in an ideal anatomical location for surgical resection, embolization can sometimes be attempted with cure in mind. One benefit of choosing an embolic approach is the myriad of embolic agents: coils, liquid embolic agents, gel foam, or microparticles. Metal coils are inserted into the nidus of the AVM or the feeding pedicles and are used to reduce the rate of blood flow to the AVM nidus. Glue or liquid embolic agents can also be injected into the AVM which once hardened blocks blood flow through the AVM nidus. Similarly, gel foam and microparticles can also be inserted into an AVM to control blood flow and reduce the risk of bleeding. There may be a financial benefit to selectively choosing cheaper particles or gel foam with or without coils for embolization that could still provide maximal benefit to the patient [8]. In those cases where surgical resection is anticipated, expensive coils and liquid embolic agents often provide no additional benefits over particles which can significantly reduce the cost of the preoperative embolization procedure.

## Conclusions

There are three primary therapeutic options for cerebral AVMs: the traditional open neurosurgical resection, stereotactic radiosurgery, and endovascular embolization. When surgical resection and radiosurgery are not options for cure by themselves, preoperative embolization can be used as adjuvant therapy to decrease bleeding risks and increase surgical success. Therefore, it is important for the cerebrovascular neurosurgeon to determine which treatment approach would optimize outcomes for their patient. Here we have presented two cases of cerebral AVMs in which pre-operative embolization followed by open surgical resection proved successful.

## References

[1] Zhang M, Connolly ID, Teo MK (2017). Management of arteriovenous malformations associated with developmental venous anomalies: a literature review and report of two cases. World Neurosurg.

[2] Abecassis IJ, Xu DS, Batjer HH (2014). Natural history of brain arteriovenous malformations: a systematic review. Neurosurg Focus.

[3] Murray G, Brau RH (2011). A 10-year experience of radiosurgical treatment for cerebral arteriovenous malformations: a perspective from a series with large malformations. Clinical article. J Neurosurg.

[4] Alaraj A, Amin-Hanjani S, Shakur SF (2015). Quantitative assessment of changes in cerebral arteriovenous malformation hemodynamics after embolization. Stroke.

[5] Narayanan M, Atwal G, Nakaji P (2017). Multimodality management of cerebral arteriovenous malformations. Handb Clin Neurol.

[6] Higgins JN, Kirkpatrick PJ (2013). Stenting venous outflow gives symptomatic improvement in a patient with an inoperable brainstem arteriovenous malformation. Br J Neurosurg.

[7] Firlik AD, Levy EI, Kondziolka D (1998). Staged volume radiosurgery followed by microsurgical resection: a novel treatment for giant cerebral arteriovenous malformations: technical case report. Neurosurgery.

[8] Ross J, Al-Shahi Salman R (2010). Interventions for treating brain arteriovenous malformations in adults. Cochrane Database Syst Rev.

